# The role of Vav3 expression for inflammation and cell death during experimental myocardial infarction

**DOI:** 10.1016/j.clinsp.2023.100273

**Published:** 2023-08-15

**Authors:** Yan Zhang, Yonglei Zhang, Qin Song, Yuanxin Wang, Jiming Pan

**Affiliations:** Department of Emergency, Yantaishan Hospital, Shandong, China

**Keywords:** Cardiomyocyte, Heart failure, Myocardial infarction, NFκB, Vav3

## Abstract

**Objectives:**

Myocardial Infarction (MI) is the leading cause of chronic heart failure. Previous studies have suggested that Vav3, a receptor protein tyrosine kinase signal transducer, is associated with a variety of cellular signaling processes such as cell morphology regulation and cell transformation with oncogenic activity. However, the mechanism of Vav3-mediated MI development requires further investigation.

**Method:**

Here, The authors established an MI rat model by ligating the anterior descending branch of the left coronary artery, and an MI cell model by treating cardiomyocytes with H_2_O_2_. Microarray analysis was conducted to identify genes with differential expression in heart tissues relevant to MI occurrence and development. Vav3 was thus selected for further investigation.

**Results:**

Vav3 downregulation was observed in MI heart tissue and H_2_O_2_-treated cardiomyocytes. Administration of Lentiviral Vav3 (LV-VAV3) in MI rats upregulated Vav3 expression in MI heart tissue. Restoration of Vav3 expression reduced infarct area and ameliorated cardiac function in MI rats. Cardiac inflammation, apoptosis, and upregulation of NFκB signal in heart tissue of MI animals were assessed using ELISA, TUNEL staining, real-time PCR, and WB. Vav3 overexpression reduced cardiac inflammation and apoptosis and inhibited NFκB expression and activation. Betulinic Acid (BA) was then used to re-activate NFκB in Vav3-overexpressed and H_2_O_2_-induced cardiomyocytes. The expression of P50 and P65, as well as nuclear P65, was significantly increased by BA exposure.

**Conclusions:**

Vav3 might serve as a target to reduce ischemia damage by suppressing the inflammation and apoptosis of cardiomyocytes.

## Highlights

• Activation of the NFκB signaling pathway aggravates myocardial injury.
• Vav3 might exert an effect in MI model by repressing the NFκB signaling pathway.
• Vav3 exerted its cardio-protective function in a NFκB-dependent manner.
• CLINICS-D-23-00277_Original Article

## Introduction

Heart failure is a progressive, multifactorial, disabling disease characterized by symptoms caused by either systolic (impaired contraction) or diastolic (impaired relaxation) ventricular dysfunction. It is the ultimate clinical condition in various cardiovascular disorders. Ischemic Heart Disease (IHD) is a major cause of death across the world.^1^ Myocardial Infarction (MI), which is caused by coronary artery occlusion, is the main cause of cardiovascular morbidity and mortality across the world.^2^ Acute MI causes decreased cardiovascular output following myocardial ischemia, cardiac surgery, and circulatory arrest in coronary heart diseases.^3^

The inflammatory response observed in MI is associated with excessive Reactive Oxygen Species (ROS) production induced by reperfusion, ultimately triggering apoptosis in the heart tissue.^4^ Inflammatory responses in the heart can be classified into pure innate immune reactions and/or a combination of innate and acquired immune reactions.^5^ The most common characteristic of the former is the production of inflammatory factors. Once myocardial ischemia occurs and results in heart failure, it usually results in both innate immune and inflammation responses.^6^ The inflammatory response and apoptosis can influence the development of MI and the repair of myocardial injury. Mild to moderate inflammation can promote myocardial repair, whereas excessive inflammatory responses can lead to secondary myocardial damage. Suppression of excessive inflammation and apoptosis are critical components of tissue repair, ventricular remodeling control, and cardiac function improvement following MI.^7^ During MI development, sensitive signal channels, such as PI3K/Akt and MAPKs, modulate transcription factors like NFκB by phosphorylation, and therefore induce the expressions of a group of redox-sensitive genes, which modulate signaling pathways including those related to inflammation, apoptosis or oxidative stress.^8^

Roth et al. demonstrated that Vav family proteins acted as key modulators as Card9/NFκB pathway in innate antifungal immunity.^9^ Nomura et al. found that reducing Vav3 expression strengthened docetaxel-triggered apoptosis by repressing androgen receptor phosphorylation under chronic hypoxia in prostate cancer cells.^10^ These reports suggest potential anti-inflammatory and apoptotic effects of Vav3. Vav family proteins (Vav1–3) are mainly expressed in hematopoietic lineage cells.^11^ Vav family proteins play a significant role in modulating T-cell activation; they facilitate cytoskeletal reorganization and are required for the mediation of cell-to-cell adhesion and lymphocyte migration.^12^ Vav3 is a Rho guanine nucleotide exchange and a cardiac-related factor.^13^ However, the involvement and detailed mechanisms of Vav3 in MI remain unknown.

The aim of this study is to assess the function of Vav3 in MI-associated inflammation and apoptosis. To achieve this goal, an MI mouse model was established through Left Anterior Descending (LAD) coronary artery ligation, and a cell model was established by H_2_O_2_ stimulation in cardiomyocytes to study the influence of Vav3 on MI-triggered myocardial injury and function loss and their relationship.

## Materials and methods

### Gene microarray

Global profiling of human genes and protein-coding transcripts was performed using Arraystar Human Gene Microarray 2.0 (Aksomics, Shanghai, China). Differentially expressed genes were identified using authoritative databases, such as Ensembl and RefSeq, based on P value, fold change, and false discovery rate. Combined analysis and hierarchical clustering were performed using in-house scripts.

### Animal study

Adult male Wistar rats (220 ± 20g) were provided by the Vital River Animal Experimental Center. The study was approved by the Ethics Committee of Yantaishan Hospital following the Chinese Guidance for Humane Laboratory Animal Use. The MI injury model was established using LAD coronary artery ligation as previously described.^14^ Briefly, the rats were intraperitoneally administered 100 mg/kg pentobarbital sodium for anesthesia. Their body temperature was maintained at 37°C using a heating pad. Subsequently, the left coronary artery was located, accessed, and ligated for 45 minutes. Reperfusion was initiated by loosening the ligature. The sham-operated control rats underwent a similar procedure without left coronary artery ligation. When reperfusion was performed, the muscle layer and skin were closed, and the rats were allowed to recover for 21 days for hemodynamic measurements. Postoperative pain was relieved by intramuscular injection of buprenorphine hydrochloride (0.65 mg/kg,).

### Experimental groups

Thirty-two healthy WT rats were randomly allocated into 4 groups with 8 rats in each group as follows: (1) Sham operation control (Sham), (2) LAD ligation (MI), (3) Lentiviral (LV)-Control Infection and LAD ligation (MI_LV-CTRL); (4) LV-VAV3 infection and LAD ligation (MI_LV-VAV3). LV-CTRL/CTNNB1 infection was induced following modeling, and the subsequent processes were the same as for the MI group.

### LV generation

The full-length VAV3 gene was cloned into the pLVX-IRES-puro vector and labeled as pLVX-VAV3 or pLVX-CTRL. HEK293T cells were subjected to triple transfection with pLVX-VAV3 and pLVX-CTRL, along with psPAX2 and pMD2.G, to obtain LV-VAV3 and LV-CTRL particles. For infection, LV particles and polybrene (5 μg/mL) were used for incubating cardiomyocytes in a growth medium. Six hours later, the medium was discarded, and the cells were used in subsequent experiments.

### Cardiac function assessment

Cardiac function was assessed as described before.^15^ Rats were intraperitoneally injected with chloral hydrate (0.3 g/kg) for anesthesia 21d after LAD surgery. The external right carotid artery was accessed, and a transducer catheter with a microtip (1.4 F) was inserted into the left ventricle. An ES 2000 model was linked to the other end. Biplane echocardiography using Vevo 2100 was used to assess the left ventricular posterior wall in systole, Left Ventricular Fractional Shortening (LVFS), and Ejection Fraction (LVEF), as previously described.^16^

### Infarct size measurement

After reperfusion for 2h, Sirius Red staining was used to measure the infarct size. In brief, the rats were sacrificed, and the hearts were fixed in paraformaldehyde overnight and cut into 5 slices (1 mm thickness), which were then embedded in flat paraffin, cut into sections (4 μm thickness), and treated with TTC staining to measure infarct volume. The slices were then placed on a light table and photographed on both sides. Subsequently, different regions were delineated. The infarct size was calculated as the infarct area volume/LV wall volume.

### ELISA

Pro-inflammatory cytokine levels in the cardiac tissue and cells were determined using as ELISA kits (Merck-Millipore, Billerica, MA, USA) following the manufacturer's instructions.

### TUNEL staining

Apoptotic cells were labeled using the TUNEL fluorescence FITC kit (Roche, Germany), as following the manufacturer's instructions. Following TUNEL staining, living, and apoptotic cell nuclei were stained by immersing heart sections (7d after MI) in Hoechst 33342 solution. Fluorescent staining was observed using a laser scanning confocal microscope (Olympus, Fluoview1000, Japan). Image-Pro Plus was used to count TUNEL-positive cells and total cell numbers.

### Real-time PCR

Total RNA was isolated from cardiomyocytes or cardiac tissue using TRIzol® reagent. cDNA was obtained by reverse transcription of RNA at 42°C for 60 min and at 75°C for 5 min. qPCR was conducted using an SYBR Green PCR Master Kit (Vazyme Biotech Co., Ltd.). The thermocycling conditions were as follows: first denaturation for 3 min at 95°C, then 40 cycles for 0.5 min at 95°C, 0.5 min at 56°C and 0.5 min at 72°C. 2^−ΔΔCq^ method was used for calculating the fold change in gene expression.^17^ The mRNA expression levels were normalized to GAPDH as an internal control. All experiments were conducted in triplicate.

### Western blot (WB)

Proteins were isolated from heart tissue and cells with RIPA lysis buffer as per relevant instructions, and a BCA protein assay kit (Beyotime Institute of Biotechnology, Shanghai, China) was used to measure the total protein concentration. The loaded proteins were subjected to SDS-PAGE and then added to Hybond-C membranes, which were then incubated overnight with the appropriate primary antibodies. The bound antibodies were visualized using horseradish peroxidase-conjugated secondary antibodies. Band intensity was measured using BandScan 5.0.

### Cardiomyocyte culture

Cardiomyocytes were collected from mice aged 6‒8 weeks and cultured.^18^,^19^ In summary, hearts were resected under sterile conditions, and ventricular specimens were cut into pieces and digested with 0.25% trypsin. The separated cells were resuspended in DMEM with 10% FBS, centrifuged (1000 rpm, 5 min), and resuspended for 120 min. The isolated cells were incubated in non-coated culture flasks. Bromodeoxyuridine (0.1 mM) was added to the medium to eliminate nonmyocytes. Cardiomyocytes were cultivated at 37°C with 5% CO_2_. Cells were exposed to 1 μM BA for 24h for BA treatment.

### Cell survival

Cell survival after H_2_O_2_ stimulation was assessed using the CCK-8 assay as per relevant instructions. Cells were inoculated into 96-well plates and then incubated for an additional 2h at 37°C after CCK-8 (10 μL) addition. OD_450nm_ (optical density) was obtained using an Infinite M200.

### Flow cytometry (FC)

Cells were cultured for 2d, and the Annexin V-FITC/PI Apoptosis Assay Kit was used to evaluate the rate of apoptosis. Cells were suspended in 1 ×  Annexin V binding buffer (100 μL), and 5 μL Annexin V and 1 μL PI were supplemented and mixed; next, the cells were incubated at RT for 15 min in the dark, and 1 ×  Annexin V binding buffer (400 μL) was supplemented to stop this process. The rate of apoptosis was measured using flow cytometry.

### Data analysis

Prism 7.0 (GraphPad Software, Inc., La Jolla, CA, USA) was used to determine the statistically significant differences, which were presented as the mean ± SD. Comparisons between two or multiple groups were performed using analysis of variance with Student's *t*-test or Tukey's posthoc test, respectively. Statistical significance was set at *p* < 0.05.

## Results

### Vav3 is downregulated in the heart tissue of MI rats

**Fig. 1:**
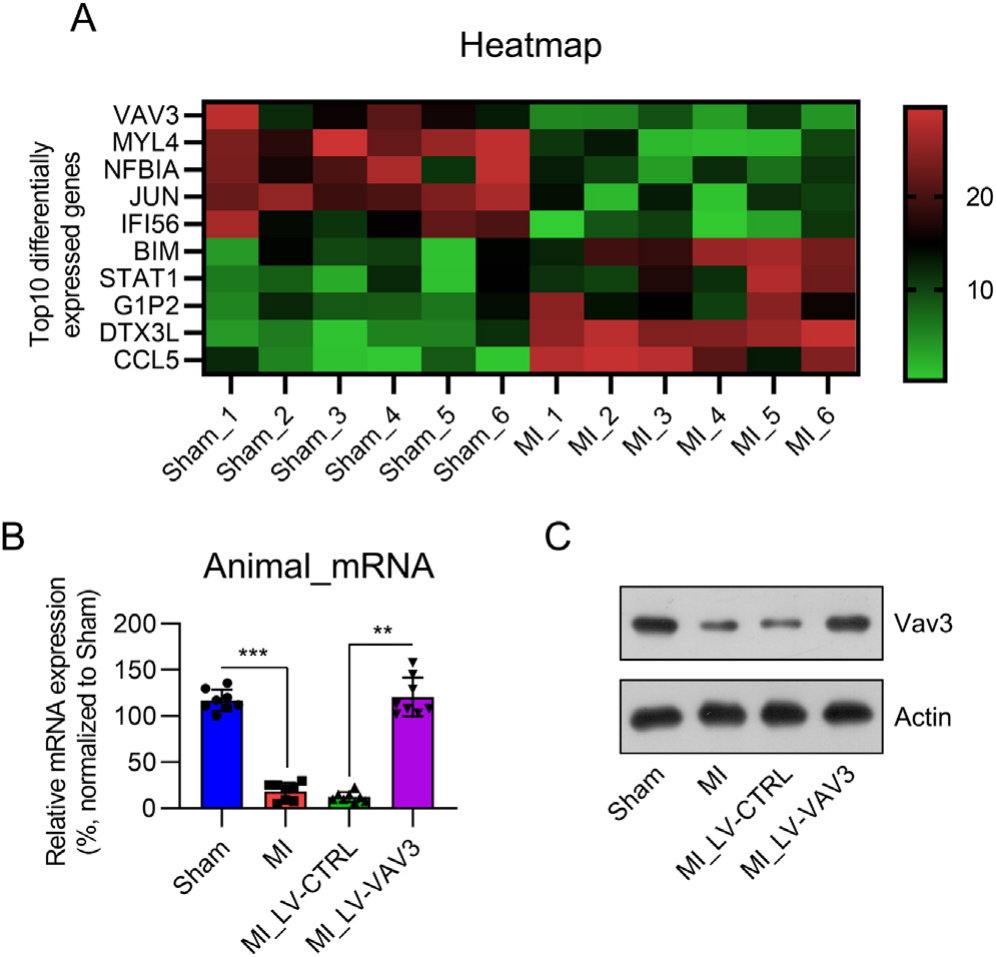
Vav3 expression in MI rat heart tissue. (A) Microarray was performed to identify the differentially expressed genes between the MI model and sham rats. Top 5 up- or downregulated genes in cluster were displayed in a heat map. (B) Real-time PCR and (C) WB was used to show Vav3 expression in the heart tissue with or without MI modelling. Results are presented as the mean ± SD of three separate experiments (n = 8; ***p < 0.001).

To identify the differentially expressed genes in MI hearts, compared with non-MI healthy heart tissue, a microarray was performed between six MI hearts from the LAD-surgery animal model and six non-MI control heart tissues (sham). Among the genes, Vav3 was the top five downregulated gene (Fig. 1A). Real-time PCR confirmed that Vav3 expression was reduced in the cardiac tissue of MI rats (Fig. 1B), while no difference in the expression of other genes was found (data not shown). WB blotting confirmed that Vav3 protein levels were lower in the MI samples than in the sham samples (Fig. 1C).

### Vav3 overexpression in cardiac tissue of MI rats

To probe the role of Vav3 in cardiac function loss and injury caused by MI modeling in rats, LV-VAV3 was injected in situ into the heart tissue of MI rats. Both real-time PCR and WB examination indicated that Vav3 expression in the heart tissue of MI rats was restored compared to that of the LV-CTRL injection group (Fig. 1B and C).

### Vav3 overexpression relieves the infarct size of MI heart tissue and cardiac function loss

**Fig. 2:**
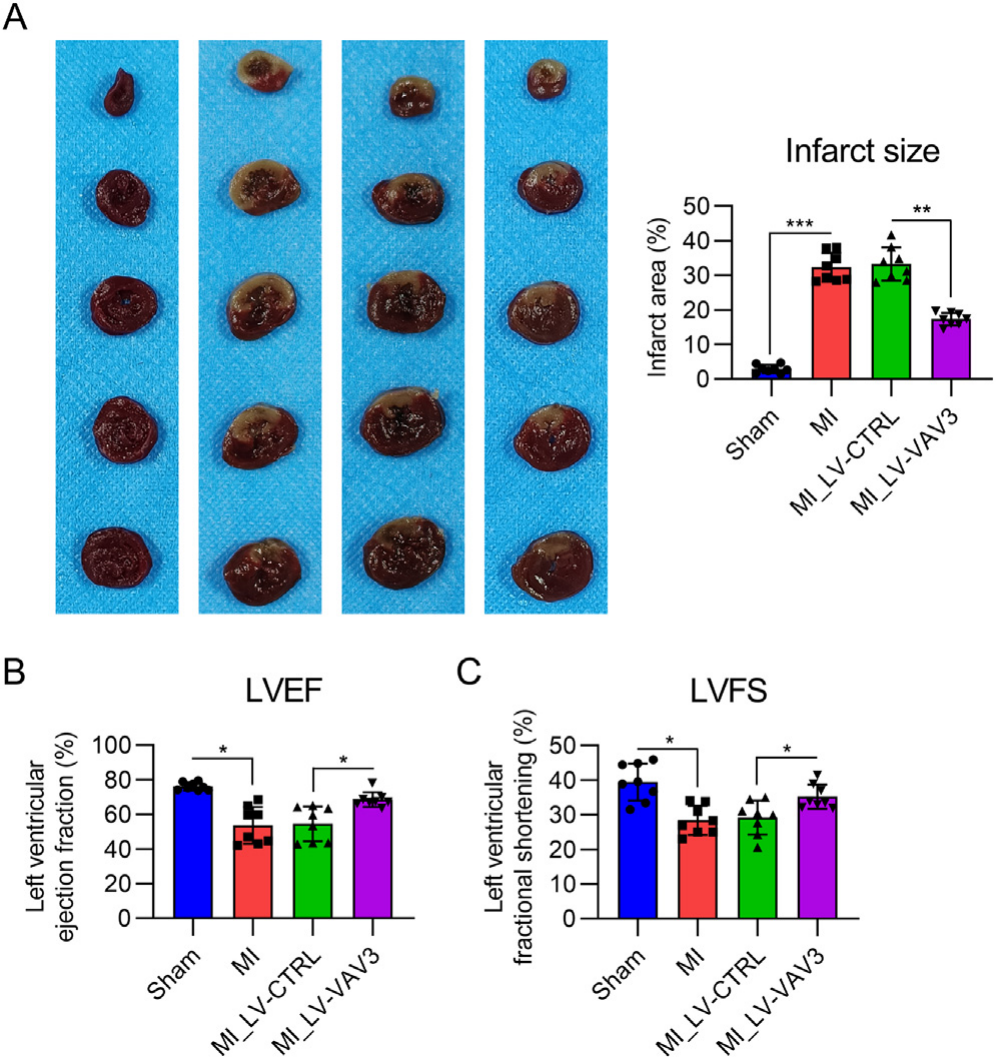
Influence of Vav3 overexpression on MI-induced infarct and Left Ventricle (LV) dysfunction in rats. (A) TTC staining was performed on the heart tissues of rats. The right panel shows the quantification of the infarct area of the heart tissue from the rats. (B) Contractile function displayed using LVEF. (C) Contractile function displayed using LVFS. Results are presented as the mean ± SD of three separate experiments (n = 8; *p < 0.05).

The authors then performed TTC staining on slides of heart samples from each group to assess the effect of Vav3 on the infarct area. TTC staining showed a significant reduction in infarct size in the MI rat model after Vav3 overexpression (Fig. 2A).

ECG was also performed to characterize the influence of Vav3 overexpression on the cardiac function loss caused by MI. LVEF and LVFS values, indicative of cardiac systolic dysfunction, were lower in the MI group than in the sham group. As expected, Vav3 overexpression alleviated cardiac systolic dysfunction, as indicated by the recovered LVEF and LVFS relative to those in the LV-CTRL group (Fig. 2B and C). These data suggest that Vav3 overexpression ameliorated MI-associated cardiac dysfunction.

### Inflammation, apoptosis, and NFκB signal activation in MI heart tissue is inhibited by Vav3

**Fig. 3:**
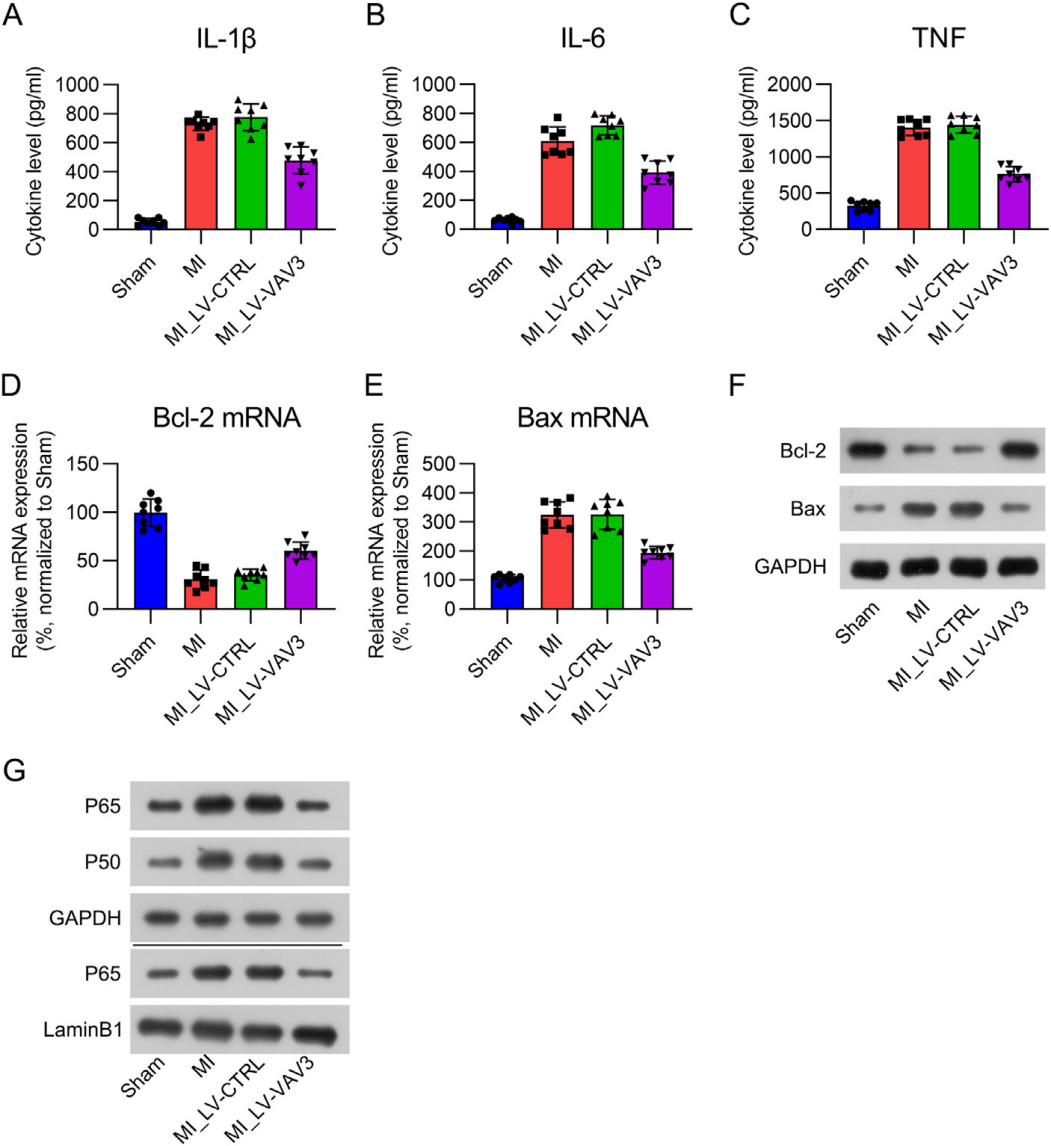
Influence of Vav3 overexpression on MI-induced inflammation, apoptosis, and NFκB in rats. (A‒C) ELISA was used to assess IL-1β, IL-6 as well as TNF levels in rat myocardial tissue homogenate. (D‒F) Real-time PCR and WB was used to show Bcl-2 and Bax expressions at mRNA and protein levels. (G) Protein levels of P50, P65, and nuclear P65 were detected by WB analysis.

Inflammation and apoptosis are two manifestations in cardiac tissue during MI,^20^ and NFκB signal is a key modulator for both. Therefore, The authors further examined the effect of Vav3 on inflammation, apoptosis, and NFκB signal activation in the heart tissue of MI rats. ELISA results indicated that three pro-inflammatory cytokines (IL-1β, IL-6, and TNF) were robustly expressed in the cardiac tissue in response to MI modeling, whereas Vav3 overexpression alleviated the production of cytokines (Fig. 3A‒C). Real-time PCR and WB of Bcl-2 and Bax expression were used to examine the apoptotic level in the hearts of MI rats. Bcl-2 mRNA and protein levels were repressed, while Bax was elevated in the heart tissue in response to MI modeling, and Vav3 overexpression reversed these changes (Fig. 3D‒F). NFκB signal activation was manifested by expression upregulation and increased nuclear location. The present data showed that the expression of P50 and P65, as well as the nuclear location of P65 was upregulated in MI hearts. However, activation of NFκB was inhibited when Vav3 was upregulated (Fig. 3G). These data suggest that Inflammation, apoptosis, and NFκB signal activation in MI heart tissue is inhibited by Vav3.

### Vav3 inhibited inflammation and apoptosis in H_2_O_2_-stimulated cardiomyocytes

**Fig. 4:**
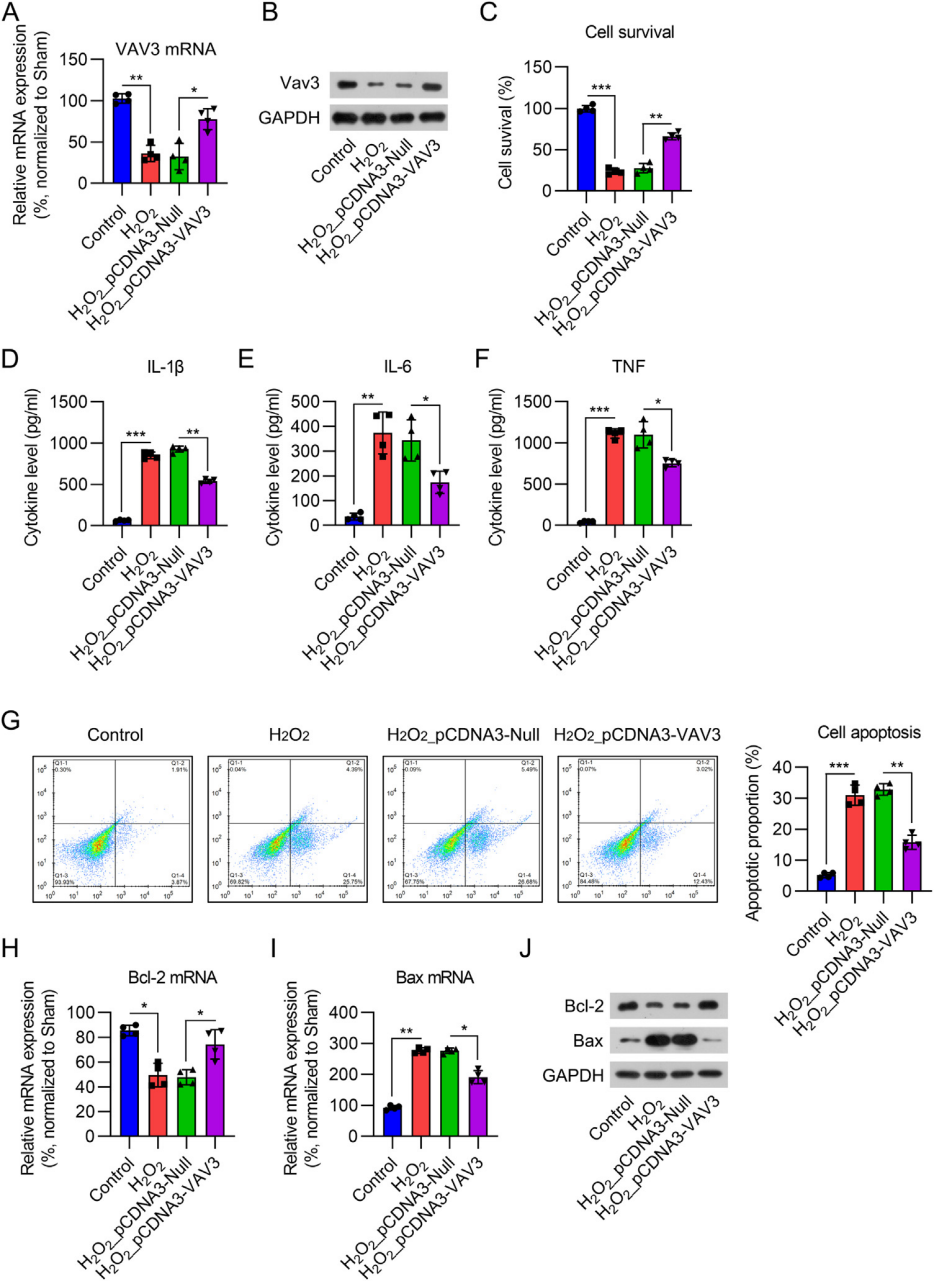
Influence of Vav3 overexpression on H_2_O_2_-triggered inflammation, apoptosis, and NFκB in cardiomyocytes. Cardiomyocytes were transfected with Vav3 overexpressed vector for 1d, and then exposed to H_2_O_2_ for 0.5h. (A) Real-time PCR and (B) WB were carried out to show Vav3 expression in cardiomyocytes. (C) CCK-8 assay was used to assess cell survival rate after different treatment and transfection. (D‒F) ELISA was conducted to measure IL-1β, IL-6 as well as TNF levels in rat myocardial tissue homogenate. (G) Apoptosis in myocardial tissue of rats was determined by FCM. (H‒J) Real-time PCR and WB was used to show Bcl-2 and Bax expressions at mRNA and protein levels.

To confirm the role of Vav3 in MI, an MI cell model was established by exposing cardiomyocytes to 12.5 μM H_2_O_2_ for 12h. Real-time PCR and WB analyses showed that Vav3 expression was reduced in cardiomyocytes after H_2_O_2_ treatment for 12h, while pre-transfection of the Vav3 overexpression vector increased its expression at both the mRNA and protein levels (Fig. 4A and B). The CCK-8 assay results suggested that the cell survival rate dramatically decreased after H_2_O_2_ induction. Vav3 overexpression increased the viability of H_2_O_2_-treated cells (Fig. 4C). Regarding inflammation, IL-1β, IL-6, and TNF-were induced by H_2_O_2_, but Vav3 reduced the generation of these three cytokines (Fig. 4D‒F). FCM indicated that the percentage of apoptotic cells increased in response to H_2_O_2_ stimulation but decreased in Vav3 overexpressed cells (Fig. 4G). Bcl-2 and Bax determination showed that Bcl-2 was reduced and Bax was upregulated in cells exposed to H_2_O_2_. As expected, Vav3 overexpression reversed the changes induced by H_2_O_2_ stimulation (Fig. 4H‒J).

### Vav3 deactivates NFκB signal in H_2_O_2_-stimulated cardiomyocytes

**Fig. 5:**
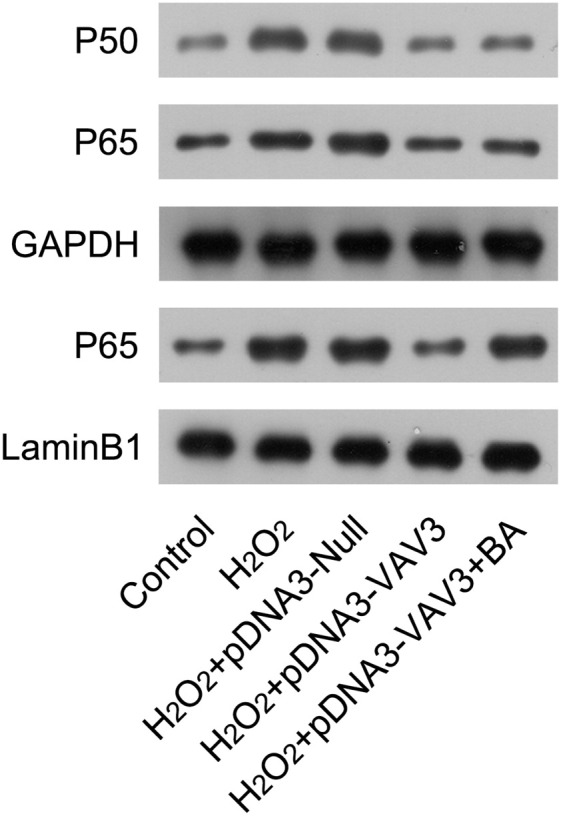
Re-activation of NFκB in Vav3 overexpressed and H_2_O_2_-stimulated cardiomyocytes. Cardiomyocytes were transfected with Vav3 overexpressed vector for 1d, and then exposed to H_2_O_2_ for 12h, followed by treatment with 1 μM BA for 24h. Protein levels of P50, P65, and nuclear P65 were detected by WB analysis.

Next, The authors examined the activation status of NFκB in cardiomyocytes in response to H_2_O_2_ induction and Vav3 overexpression. In H_2_O_2_-treated cardiomyocytes, the expression of P50 and P60, as well as nuclear P65, was upregulated compared with non-treated cells. After Vav3 overexpression, levels of P50, P65, and nuclear P65 were reduced (Fig. 5), suggesting an inhibitory role of Vav3 on NFκB activation. To elucidate the involvement of NFκB in Vav3-regulated inflammation and apoptosis of the MI cell model, the cardiomyocytes with H_2_O_2_ exposure and Vav3 overexpression were treated with BA, a NFκB activator, to re-activate NFκB. The present results showed that although the expression of P50 and P65 was not altered by BA administration, the nuclear localization of P65 was significantly improved (Fig. 5).

### BA treatment counteracts the effect of Vav3 overexpression in H_2_O_2_-stimulated cardiomyocytes

**Fig. 6:**
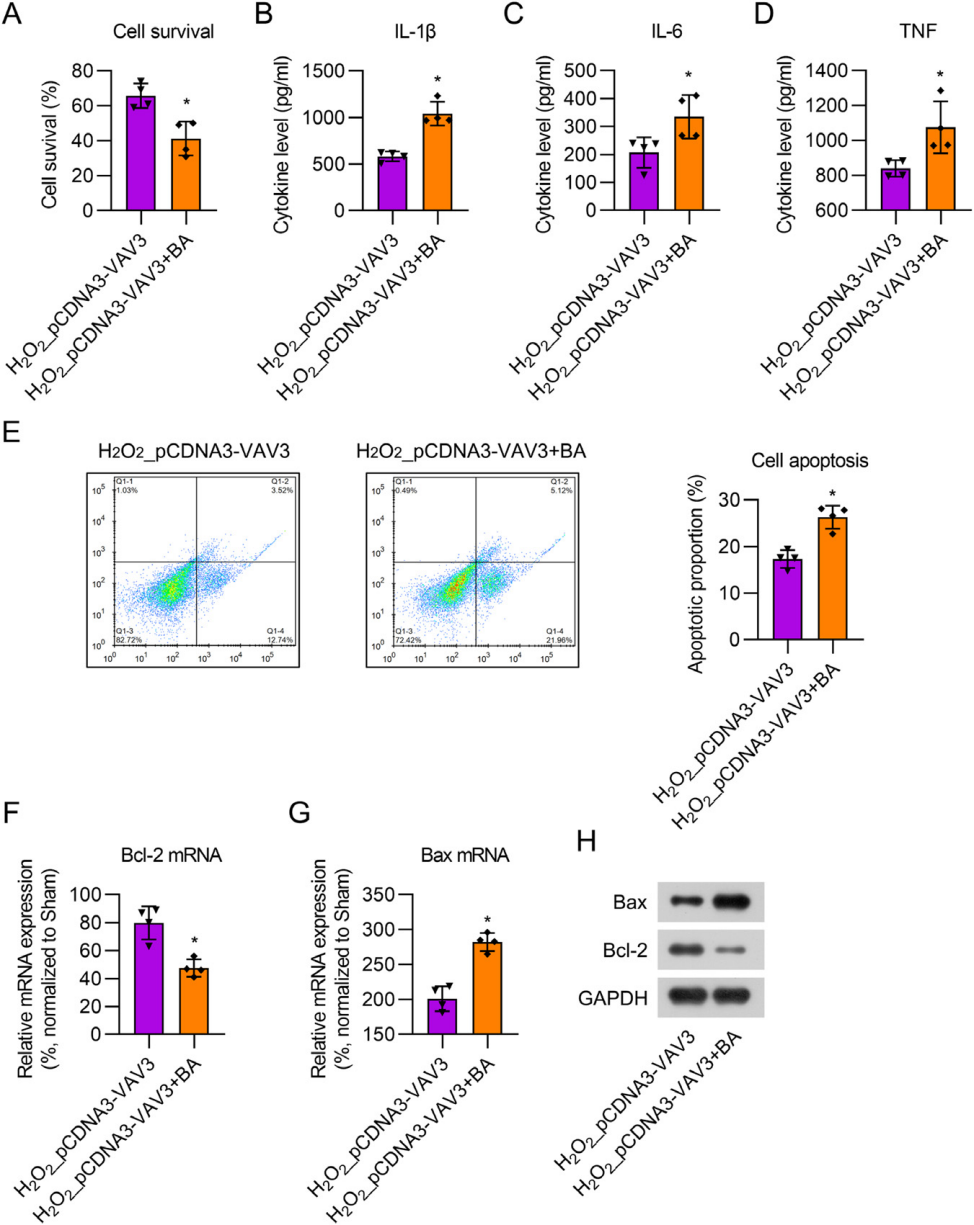
BA exposure counteracted the influence of Vav3 overexpression on inflammation and apoptosis in H_2_O_2_-treated cardiomyocytes. (A) CCK-8 assay was used to assess cell survival rate after different treatment and transfection. (B‒D) ELISA was used for measuring IL-1β, IL-6 as well as TNF levels in rat myocardial tissue homogenate. (E) Apoptosis in myocardial tissue of rats was determined by FCM. (F‒H) Real-time PCR and WB examination displayed Bcl-2 and Bax expressions at mRNA and protein levels.

To evaluate the effect of BA, cardiomyocytes with Vav3 overexpression were first treated with 12.5 μM H_2_O_2_ for 12h, followed by treatment with 1 μM BA for 24h. CCK-8 assay showed that BA treatment reduced cell viability in Vav3 overexpressed H_2_O_2_-stimulated cells (Fig. 6A). ELISA results indicated that the secretion of cytokines was dramatically upregulated in the BA-treated cell supernatant compared to that in the non-BA treatment group (Fig 6B‒D). FCM data showed that the number of apoptotic cells significantly increased following BA administration (Fig. 6E). Real-time PCR and WB showed that BA administration reversed the effect of Vav3 overexpression on Bcl-2 and Bax expression in H_2_O_2_-stimulated cardiomyocytes (Fig 6F‒H). Taken together, the present results demonstrate that BA treatment counteracts the influence of Vav3 overexpression on inflammation and apoptosis in H_2_O_2_-stimulated cardiomyocytes.

## Discussion

MI is the leading cause of death worldwide. The common standard therapy is to perform reperfusion as soon as possible. Nevertheless, reperfusion can paradoxically trigger severe damage to the myocardium, such as cytokine secretion, neutrophil infiltration, calcium overload, and ROS overgeneration. Experimental research and clinical trials have not yet identified efficient therapies to protect the myocardium against reperfusion injury. Therefore, the present study aimed to identify new targets in heart failure therapy to minimize the extent of MI injury. The authors investigated whether Vav3 protected the myocardium in LAD surgery-induced MI rats and H_2_O_2_-treated cardiomyocytes via regulating the NFκB signaling pathway.

In the Vav family, Vav3 plays a critical role in tumor development and metastasis.^21^ Numerous studies have focused on the influence of Vav3 on the development and tumorigenesis in lymphoma, bladder, and breast cancers and indicated that Vav3 expression is increased in these cancers.^22^, ^23^, ^24^ Vav3 regulates various signaling pathways by regulating the activity of Rho family members. The main downstream signaling pathways influenced by Vavs are MAPK and PI3K-Akt.^10^,^25^ In the present study, by comparing differentially expressed genes in heart tissues between MI and sham-operated rats, The authors observed that Vav3 expression was downregulated in MI hearts. Further validation by real-time PCR and WB analyses confirmed that Vav3 expression was lower in the MI group than in the sham group. In vitro cell-based experiments also indicated that Vav3 expression decreases in cardiomyocytes in response to H_2_O_2_ stimulation. Further investigations suggested that Vav3 exerts cardioprotective effects against MI.

Inflammation plays a critical role in cardiac remodeling and heart failure following MI.^26^, ^27^, ^28^ The inflammatory response that occurs following tissue damage is a critical physiological process in healing; however, an excessive response can generally repress fibrosis repair. MI is accompanied by increased cardiomyocyte apoptosis. Myocardial tissue apoptosis in rats in the MI group was dramatically increased, and the ratio of Bax to Bcl-2 was upregulated.^29^ Therefore, The authors evaluated the impact of Vav3 on pro-inflammatory cytokines by ELISA and apoptosis by TUNEL, FCM, Bax, and Bcl-2 expression. The results of this study indicated that inflammation and apoptosis were notably upregulated following MI, which is consistent with previous studies. the enhancement of pro-inflammatory cytokines and the proportion of apoptotic cells after MI was notably reduced after Vav3 overexpression, suggesting that Vav3 has anti-inflammatory and anti-apoptotic effects following MI.

The key element in this inflammatory response and apoptosis is the activation of the nuclear NFκB family.^30^ NFκB has been considered a redox-sensitive transcription factor relevant to immune reactions. Activation of the NFκB signaling pathway aggravates myocardial injury.^29^ The NFκB-relevant protein levels of P50 and P65 and nuclear P65 were dramatically elevated following MI. After activation, NFκB translocates to the nucleus to modulate pro-inflammatory cytokine transcription.^31^ In this study, in vitro and in vivo experiments both confirmed that NFκB signal was activated in response to MI modeling and H_2_O_2_ treatment. Re-activation of NFκB was exerted by treating the cells with BA. Further investigation showed that BA administration significantly restored the excessive inflammation and apoptosis caused by Vav3 overexpression, suggesting that Vav3 exerted its cardio-protective function in an NFκB-dependent manner.

## Conclusion

The present data suggested that Vav3 might exert an effect in the MI model by influencing inflammation and apoptosis via repressing the NFκB signaling pathway. Vav3 might serve as a target to reduce ischemia damage by suppressing the inflammation and apoptosis of cardiomyocytes.

## Authors' contributions

Yan Zhang and Jiming Pan conceived the study and designed the experiments. Yonglei Zhang, Qin Song and Yuanxin Wang contributed to the data collection, performed the data analysis, and interpreted the results. Yan Zhang wrote the manuscript. Jiming Pan contributed to the critical revision of the article. All authors read and approved the final manuscript.

## Funding

This research did not receive any specific grant from funding agencies in the public, commercial, or not-for-profit sectors.

## Declaration of Competing Interest

The authors declare no conflicts of interest
